# Highly sensitive feature detection for high resolution LC/MS

**DOI:** 10.1186/1471-2105-9-504

**Published:** 2008-11-28

**Authors:** Ralf Tautenhahn, Christoph Böttcher, Steffen Neumann

**Affiliations:** 1 Leibniz Institute of Plant Biochemistry, Department of Stress and Developmental Biology, Weinberg 3, 06120 Halle, Germany

## Abstract

**Background:**

Liquid chromatography coupled to mass spectrometry (LC/MS) is an important analytical technology for e.g. metabolomics experiments. Determining the boundaries, centres and intensities of the two-dimensional signals in the LC/MS raw data is called feature detection. For the subsequent analysis of complex samples such as plant extracts, which may contain hundreds of compounds, corresponding to thousands of features – a reliable feature detection is mandatory.

**Results:**

We developed a new feature detection algorithm *centWave *for high-resolution LC/MS data sets, which collects regions of interest (partial mass traces) in the raw-data, and applies continuous wavelet transformation and optionally Gauss-fitting in the chromatographic domain. We evaluated our feature detection algorithm on dilution series and mixtures of seed and leaf extracts, and estimated recall, precision and F-score of seed and leaf specific features in two experiments of different complexity.

**Conclusion:**

The new feature detection algorithm meets the requirements of current metabolomics experiments. *centWave *can detect close-by and partially overlapping features and has the highest overall recall and precision values compared to the other algorithms, *matchedFilter *(the original algorithm of *XCMS*) and the centroidPicker from *MZmine*. The *centWave *algorithm was integrated into the Bioconductor R-package *XCMS *and is available from

## Background

Metabolomics aims at the unbiased and comprehensive quantification of metabolite concentrations in organisms, tissues, or cells [1,2]. The combination of chromatographic separation with subsequent mass spectrometric detection has emerged as a key technology for multiparallel analysis of low molecular weight compounds in biological systems. Gas chromatography-mass spectrometry (GC/MS) based techniques are mature and well-established, but restricted to volatile compounds, often requiring chemical derivatisation. High-performance liquid chromatography-mass spectrometry (HPLC/MS) facilitates the analysis of compounds of higher polarity and lower volatility in a much wider mass range without derivatisation [3-5]. With LC/MS the injected sample is separated on the chromatographic column, resulting in the consecutive elution of different compounds. The mass spectrometer acquires mass spectra from the column output at a specified scan rate, so each compound can be measured in several consecutive scans. Due to the fact that each eluting compound gives rise to a number of mass signals (adducts, fragments and isotopic peaks), a metabolite induces several two-dimensional features.

In the following, we use the term "feature" for a bounded, two-dimensional (*m/z *and retention time) LC/MS signal. The term "peak" is used for one-dimensional signals: both *m/z *peaks (centroids) in the mass spectrum and chromatographic peaks.

For complex metabolomics samples, the LC/MS data contains hundreds to thousands of metabolites. For the statistical analysis of biological experiments the feature intensity is of interest and has to be calculated from the raw data. Spectra can be acquired in profile mode or centroid mode. Vendor supplied centroidisation algorithms usually employs machine-specific models, which are superior to generic approaches. In addition, the centroid mode results in considerable size reduction of the LC/MS data set.

The processing pipeline for LC/MS based metabolomics can be divided into the following steps:

1. Signal preprocessing and centroidization in *m/z*,

2. Two-dimensional feature detection and integration

3. Alignment of corresponding features in multiple samples

4. Statistical analysis, chemical and biological interpretation.

Feature detection is a crucial step in the LC/MS data processing pipeline – it should be reliable, i.e. report as many as possible "real" features, while keeping the false positive rate low. The challenge for the algorithms is to detect features of low intensity induced by compounds with low abundance on the one hand, and to avoid feature-like signals caused by e.g. chemical noise on the other hand.

Several frameworks for feature detection (and alignment) of metabolomics LC/MS data have been developed in the last years, both commercial products such as MarkerLynx (Waters), the closed-source (but freely-available) MetAlign [6], or XCMS [7] and *MZmine *[8] which have open-source licenses. Other packages, some of them specific for LC/MS-based proteomics, have been reviewed in [9].

A widely used approach for the processing of LC/MS data is to transform the raw data into a matrix representation with the dimensions *m/z*, retention time and intensity. To convert high resolution mass spectra into this representation, it is necessary to divide the *m/z *axis into equidistant chunks depending on the resolution of the mass spectrometer, e. g. 0.1 *m/z *wide. This procedure is usually referred to as binning. Some drawbacks of this method were already mentioned in [7,10,11]. In particular, specifying the optimal bin size for the particular data set can be difficult. If the bin size is chosen too small, chromatographic peaks are alternating between bins and cannot be detected due to the loss of the chromatographic shape. If the bin size is too large, peaks can overlay each other and small features are rather buried by the increased chromatographic noise level. On the positive side it should be mentioned that the binning approach is all-purpose and allows for a fast data processing.

A density based LC/MS feature detection approach – an alternative to the common binning technique – was introduced by Stolt et al. [10]. The authors consider the emerging analyte as a region of data points with high density anked by a specific "data void". Based on these properties, they calculate a potential field which is then used to create a matrix of mass traces (runtime ~2 h/sample). Recently, the extraction of "pure ion chromatograms" using Kalman tracking was demonstrated in [11]. The applicability of Wavelet based techniques for peak picking in MALDI- and SELDI-TOF mass spectra was shown by e.g. [12-15]. Here we will discuss a new method for the reliable detection and integration of two-dimensional LC/MS signals, referred to as features. By using a combination of a density based technique to detect regions of interest in the *m/z *domain, and a Wavelet based approach to resolve chromatographic peaks, we achieve a high sensitivity even in very complex mixtures compared to two other algorithms, *matchedFilter *(the original algorithm of *XCMS*) and the *centroidPicker *from *MZmine*.

So far, there is no common method for evaluating the performance of feature detection algorithms. Even for the same feature detection algorithm, different parametrisation can lead to (vastly) different results, if e.g. many false positive noise signals are detected as features. Therefore the absolute number of detected features per sample is not suitable to characterise a feature detection algorithm. More elaborate approaches consider mixtures of known compounds spiked into complex samples [16]. To the best of our knowledge, no evaluation has been performed to assess recall and precision of feature detection algorithms for multiple complex samples.

The remainder of this paper is structured as follows: In section 2 we give a detailed description of the *centWave *algorithm, followed by the description of the experimental comparison between several feature detection algorithms. In section 3 we present the evaluation results and discuss the benefits of *centWave*, followed by a conclusion and outlook of expected future developments in section 4.

## Methods

This section describes the *centWave *method which combines density based detection of regions of interest in the *m/z *domain, and a Continuous Wavelet Transform (CWT) based approach for chromatographic peak resolution. The experimental setup is depicted as well as the layout of the evaluation procedure.

### 2.1 The centWave algorithm

#### 2.1.1 Detecting regions of interest (ROI) in the m/z domain

**Figure 1:**
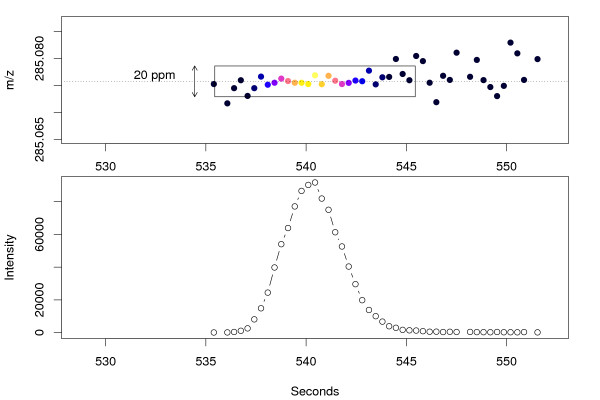
**Mass trace and chromatographic peak of Biochanin A [*M *+ *H*]^+ ^mass signal**. The upper panel shows the mass trace of the biochanin A [*M *+ *H*]^+ ^mass signal across 10 seconds with colour-coded intensities. The corresponding chromatographic peak is shown below.

To circumvent the mentioned problems of the binning technique, an alternative, fast computing approach was used which directly detects regions of interesting mass traces. Figure 1 shows the extracted ion chromatogram and the corresponding *m/z *centroids in the consecutive mass spectra for a typical LC/MS feature, recorded in centroid mode. With the chromatographic peak emerging, the consecutive centroids form a compact mass trace bounded in *m/z *and retention time. The *m/z *deviation is determined by the mass accuracy of the mass spectrometer and typically increases with lower signal intensities.

Due to the fact that the mass accuracy (*μ*, given in ppm) of the mass spectrometer and the minimum chromatographic peak width is known or can easily be assessed, it is possible to directly scan for regions where at least p_min _centroids with a deviation less than *μ *ppm occur. This task is achieved by the following algorithm for samples in centroid mode, with scans numbered *s *= 1,...,*S*:

1. Initialisation:

(a) Initialise a list ROI using all *m/z *values ${\text{mz}}_{i}^{s}$ from the first scan:

∀ *i *= 1,..., *N*, *N *= |mz^*s *= 1^|: ROI(*i*).values(1) = ${\text{mz}}_{i}^{s=1}$

(b) Initialise the *m/z *mean value for each actually processed region :

ROI(*i*).mzmean = ${\text{mz}}_{i}^{s=1}$, *i *= 1,..., *N, N *= |mz^*s *= 1^|

2. For each scan *s *= 2,..., *S *:

(a) For each *m/z *value ${\text{mz}}_{i}^{s}$, *i *= 1,..., *N, N *= |mz^*s*^| in the current scan *s*:

Exists *j, j *= 1,..., *J, J *= |ROI| such that |ROI(*j*).mzmean - ${\text{mz}}_{i}^{s}$| < = *μ *?

• **Yes**: Append ${\text{mz}}_{i}^{s}$ to ROI(*j*) and update the *m/z *mean value

*K *= |ROI(*j*).values| + 1, ROI(*j*).values(*K*) = ${\text{mz}}_{i}^{s}$

ROI(*j*).mzmean = $\frac{1}{K}\sum_{k=1}^{K}ROI(j)$.values(*k*)

• **No**: Initialise a new ROI and append it to the list

*J *= |ROI| + 1, ROI(*J*).values(1) = ${\text{mz}}_{i}^{s}$, ROI(*J*).mzmean = ${\text{mz}}_{i}^{s}$

(b) Check & Cleanup:

• Remove all ROI which were not extended in step 2a *and *contain less than p_min _centroids

• Mark ROI that were not extended, but contain at least p_min _centroids as completed

Optionally an intensity filter (*prefilter *= (*k*, *I*), e. g. *prefilter *= (2, 100)) can be set to early discard regions of small intensity. Then only those ROI are retained (in step 2b) that contain at least *k *consecutive values with intensity ≥ *I*. This prefilter vastly speeds up the overall processing time.

**Figure 2:**
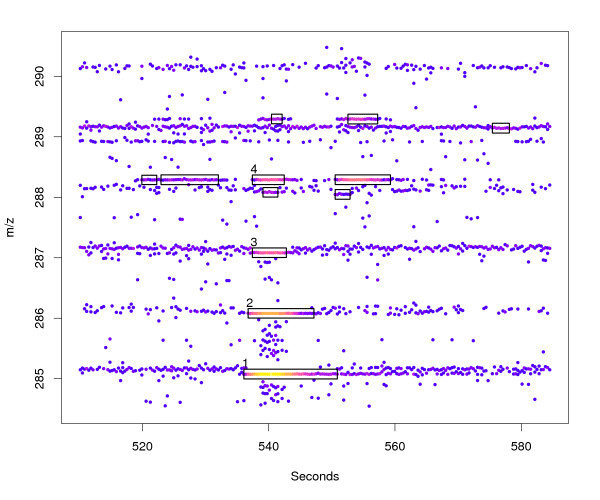
**Region Of Interest (ROI) detection**. Raw data in the chromatographic and *m/z *region around the [*M *+ *H*]^+ ^mass signal (1) of biochanin A. In addition to the three isotopic peaks (2–4) other mass signals are marked as ROIs.

Each *m/z *value needs to be considered only once, so the ROI algorithm is fast (approximately 10–20 seconds on a 2.5 GHz CPU for a measurement with 3000 scans). Figure 2 shows the result of the ROI detection algorithm for a small region of a complex LC/MS sample.

In some rare cases "gaps" are observed in the mass trace of features with low intensity. Due to the fact that each ROI is laterally extended for the following chromatographic peak detection, only a small contiguous region needs to be found for the successful detection of such features. To a certain extent, the algorithm is therefore able to detect features with such gaps. Otherwise, in case of samples which might show this phenomenon more often, the algorithm can easily be modified to be even more "gap-tolerant". In contrast to binning, this approach has the advantage that no fixed bin size has to be chosen. Each ROI is detected separately and the drawbacks of binning can be circumvented. Unlike binning the result is not a matrix but a list of mass traces with different lengths. Depending on the chromatography and the mass accuracy of the mass spectrometer, each ROI may contain none, exactly one or more than one distinct chromatographic peaks. Therefore it is necessary to subject each ROI to an extensive analysis in the chromatographic domain.

#### 2.1.2 Detecting chromatographic peaks

**Figure 3:**
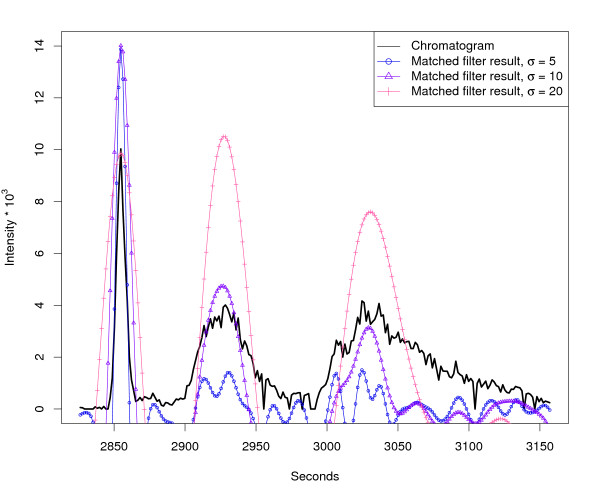
**Matched filter effects, example region 1**. HPLC/ESI-QTOF-MS of a *A. thaliana *leaf extract. Extracted ion chromatogram (277.213 – 277.221 *m/z*) and matched filter results using second derivative Gaussian with different filter widths. Negative filter values were omitted.

Depending on the separation technique (e. g. HPLC/UPLC/CE) features can show considerable variations in their chromatographic width and shape. The matched filter approach makes use of a filter based on a model peak with defined shape and fixed width. This technique gives good results in most cases and was shown to work in principle also for peaks of differing width and shape (see [17,18]) but nevertheless some problems occur if the model peak width is not chosen appropriately. Figure 3 shows a mass trace from a HPLC/MS sample, containing three peaks of different width. The application of three independent matched filters with different width of the model peak (second derivative Gaussian) reveals the problem of assessing the perfect model peak width. Narrow peaks are found perfectly with a small model peak width (e. g. *σ *= 5–10 s) while broad peaks can only be properly detected with an increased model peak width (e. g. *σ *= 20 s).

**Figure 4:**
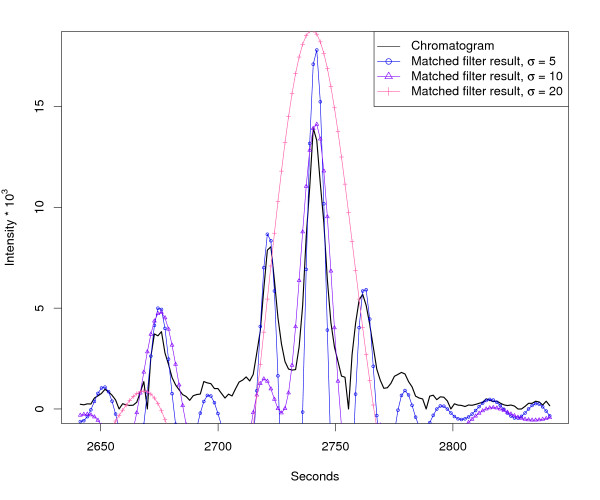
**Matched filter effects, example region 2**. HPLC/ESI-QTOF-MS of a *A. thaliana *leaf extract. Extracted ion chromatogram (967.53–967.56 *m/z*, same sample that was used for Figure 3) and matched filter results using second derivative Gaussian with different filter widths. Negative filter values were clipped.

**Figure 5:**
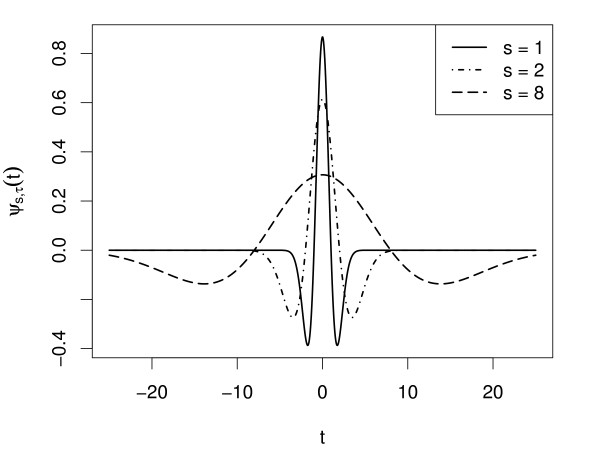
**Mexican Hat Wavelet**. Mexican hat wavelet at different scales.

Another aspect of this optimisation problem are chromatographic close-by peaks. Figure 4 shows the response of three independent matched filters with different *σ *on a chromatogram with many narrow, close-by peaks. It can be seen that only a matched filter with a very small model peak width (e. g. *σ *= 5 s) gives reasonable results in this case. Figure 3 and 4 are examples from the same LC/MS measurement. In this case, none of the three chosen model peak widths yields satisfying results for all occuring peaks. The enhancement of the matched filter approach is the peak detection on multiple scales using Continuous Wavelet Transform (CWT), which reliably detects chromatographic peaks of differing width. The CWT is widely used in signal processing and pattern recognition. The mathematical representation [19] is as follows:

$$\begin{array}{c} (T^{\text{wav}}f)(s,\tau )=\int_{-\infty }^{\infty }f(t)\psi_{s,\tau }(t)dt\\ \begin{array}{cc} \psi_{s,\tau }(t)=\frac{1}{\sqrt{s}}\psi \left(\frac{t-\tau }{s}\right), & \begin{array}{cc} s\in {\mathbb{R}}^{+}-\{0\}, & \tau \in \mathbb{R} \end{array} \end{array} \end{array}$$

where *f*(*t*) is the signal, *ψ *the mother wavelet, *s *the scale and *τ *the translation. The result of the CWT is a two-dimensional matrix of wavelet coefficients *T*^wav^. Since the "Mexican Hat" wavelet (normalised second derivative of Gaussian $e^{-x^{2}/2}$, Figure 5) is used as the mother wavelet, the result of the CWT is comparable to the combined application of the matched filter technique with the second derivative Gaussian of different widths as model peak. The algorithms for CWT and CWT-coefficient analysis described and implemented in [13] for the peak detection in SELDI/TOF spectra were adapted for peak detection in the chromatographic domain.

#### 2.1.3 The centWave workflow

The three relevant input parameters for the *centWave *algorithm are

1. Mass deviation *μ *in ppm, typically set to a generous multiple of the mass accuracy of the mass spectrometer. We use *μ *= 30 ppm for the Bruker MicrOTOF-Q, which is advertised with a mass accuracy of 3–5 ppm.

2. Chromatographic peak width range w_min_, w_max _in seconds, e. g. w_min_, w_max _= (5, 10) for UPLC separation as described in the experimental setup.

3. Signal to noise ratio threshold SNR_Thr_, e.g. SNR_Thr _= 10

The following is the description of the most important steps of the *centWave *workflow:

• The scale range s_min_, s_max _for the CWT and the p_min _parameter for the ROI detection are calculated from the input parameters w_min_, w_max _and the average inter-scan distance.

• ROI detection (see section 2.1.1) is performed using the parameters *μ *and p_min_

• Chromatographic analysis of each detected ROI:

- To accommodate noise and baseline estimation, each ROI is laterally extended by a multiple of the expected chromatographic peak width

- Local noise and baseline estimation: Let *x *be the vector of intensity values of the actual (extended) ROI, and *x*_*t *_the 10% trimmed *x *(5% of the smallest and 5% of the largest intensity values are discarded). Then the baseline BL is assessed as the mean value of *x*_*t *_and the noise level NL as the standard devation of *x*_*t*_.

- The Continuous Wavelet Transform (see 2.1.2) is applied to the intensity values of the ROI (the extracted ion chromatogram), using the scale range s_min_,..., s_max_.

- Local maxima of the CWT coefficients at each scale are detected.

- "Ridges" can be identified by linking the detected local maxima (described in [13]). The ridges describe the scale range where the chromatographic peak was located. If more than one chromatographic peak was detected, the following steps are applied for each peak separately.

- Locate the chromatographic peak boundaries rt_min _and rt_max _by descent on the filtered peak data, i.e. the CWT coefficients of the scale where the peak was optimally located.

- Calculate the feature intensity I using the intensity values within rt_min _and rt_max_. I_max _is defined as the maximal intensity value within this range.

- Compute the *m/z *centroid of the feature as the weighted mean of the *m/z *values within rt_min _and rt_max_.

- Calculate the signal to noise ratio SNR = (I_max _– BL)/NL of the feature. Discard the feature if SNR < SNR_Thr_.

- The deviation μ* of *m/z *values within rt_min _and rt_max _is calculated in ppm. The value μ* can be interpreted as the mass deviation value which would have been sufficient for the detection of this feature.

- Optionally, a Gaussian curve is fitted to the feature, using the Nonlinear Least Squares (NLS) implementation of R.

**Figure 6:**
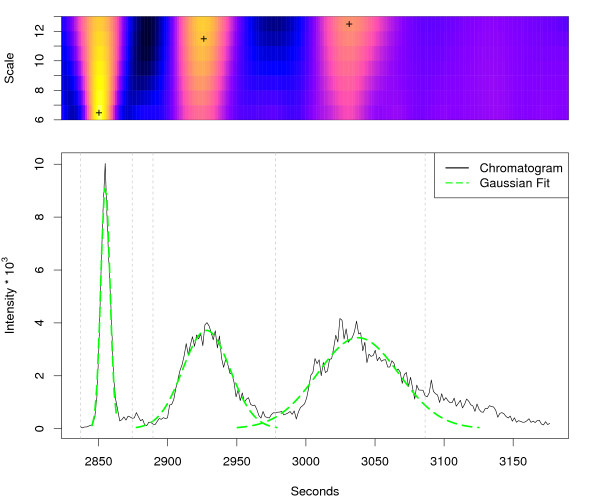
**centWave results for example region 1**. *centWave *results for example region 1. The lower part shows the same extracted ion chromatogram (277.213–277.221 *m/z*) as in Figure 3 and the detected chromatographic peaks from the *centWave *algorithm as Gaussian fits. The upper part shows the CWT coefficients on the different scales. A cross marks the scale where the peak was optimally localised. The vertical grey lines show the peak borders which were estimated from the coefficients of this scale.

**Figure 7:**
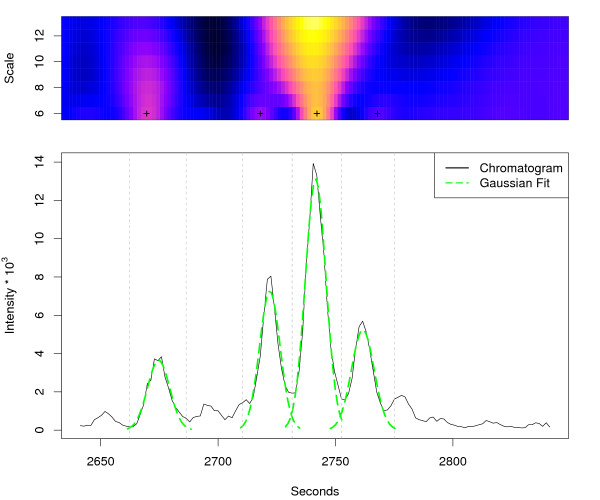
**centWave results for example region 2**. *centWave *results for example region 2. The lower part shows the same extracted ion chromatogram (967.53–967.56 *m/z*) as in Figure 4 and the detected chromatographic peaks from the *centWave *algorithm as Gaussian fits. The upper part shows the CWT coefficients on the different scales. A cross marks the scale where the peak was optimally localised. The vertical grey lines show the peak borders which were estimated from the coefficients of this scale.

The result of the *centWave *algorithm for the regions shown in Figure 3 and 4 is depicted in Figure 6 and 7, respectively. The following experiments were designed to pose challenges with increasing complexity to the feature detection algorithms. We used complex mixtures with *Arabidopsis thaliana *leaf and seed extracts.

### 2.2 Experimental setup and Sample description

*Arabidopsis thaliana *(ecotype Col-0) was grown under controlled conditions and pooled after harvest. Methanolic extracts were prepared from ground seed and leaf tissue. o-Anisic acid, biochanin A, p-coumaric acid, ferulic acid, *N*-(3-indolylacetyl)-L-valine, kinetin, indole-3-acetonitrile, indole-3-carbaldehyde, kaempferol, phloretin, phlorizin and phenylglycine, rutin, and phenylalanine-d5 were used as marker compounds. The chromatographic separations were performed on an Acquity UPLC system (Waters) equipped with a modified *C*_18 _column with a 20 min water/acetonitrile gradient. The eluted compounds were detected by a Bruker MicrOTOF-Q in positive ion mode at a scan rate of 3 Hz. Mass calibration was performed against lithium formiate. The detailed experimental setup is available as Additional file 1.

**Sample 1 **A mixture containing each of the fourteen marker compounds (referred to as MM14) at a concentration of 20 *μ*M was prepared and analysed by UPLC/ESI-QTOF-MS.

**Sample set 2 **Mixtures containing solvent and seed or leaf extracts were prepared with following volume portions (solvent/seed/leaf, v/v/v): 0/100/0, 25/75/0, 50/50/0, 75/25/0, 0/0/100, 25/0/75, 50/0/50, 75/0/25. The sample set (8 samples) was analysed by UPLC/ESI-QTOF-MS in ten technical replications.

**Sample set 3 **Mixtures containing solvent, seed, and leaf extracts were prepared with following volume portions (solvent/seed/leaf, v/v/v): 75/0/25, 0/75/25, 0/50/50. The sample set (3 samples) was analysed by UPLC/ESI-QTOF-MS in ten technical replications.

All files were acquired in centroid mode and converted to mzData file format using Bruker CompassXport software. The data sets are available at .

### 2.3 Parameter optimisation

Beside *centWave*, there are currently only two other feature detection algorithms available [9], which meet the following criteria: freely available, open source, and suited for feature detection in metabolomic LC/MS samples measured in centroid mode: *matchedFilter*- the originally implemented algorithm from XCMS and the *centroidPicker *from *MZmine *(Table 1).

**Table 1:** Overview of the evaluated feature detection algorithms

Algorithm	Framework	Version	Programming Language	Availability
centroidPicker	MZmine	0.60	Java	

centWave matchedFilter	XCMS	1.12.1	C, R	

Overview of the compared feature detection algorithms for metabolomics data. MZmine has three feature detection algorithms implemented, but only the centroidPicker is suitable for centroided data and therefore was used for the evaluation.

The three algorithms tested have a number of parameters each, which have to be tuned to deliver good performance on the analytical setup. The *centWave *algorithm uses the *peakwidth *(= w_min_, w_max_) parameter to specify the chromatographic peak width range, the *ppm *parameter to set the tolerated mass deviation and *snthresh*, which defines the chromatographic signal-to-noise threshold. The *matchedFilter *algorithm has a similar parameter *snthresh*, the chromatographic peak width is specified by the *fwhm *parameter, which defines the width of the model peak for matched filtering. The mass accuracy is indirectly defined by the bin size (parameter *step*).

The centroidPicker from *MZmine *also needs a bin size to be specified (*bin size*), and additionally the tolerated mass deviation (*m/z tolerance*). Moreover, there are five parameters that affect the chromatographic domain: *chromatographic threshold level*, *intensity tolerance*, *minimum peak duration*, *minimum peak height *and *noise level*. The first two of those are specified as a relative value, while the last three are set using absolute values.

The parameters of the three algorithms were tuned to detect as many of the real features, without allowing too many false positives. Based on known "good working" settings, we performed parameter sweeps and evaluated the number of real features and the number of other ("false") features for each setting. After initial optimisation of the other parameters, we found that for *centWave *and *matchedFilter *the *snthresh *parameter shows the highest influence on this ratio.

The centroidPicker from *MZmine *was more complex to optimise, due to its many parameters. Using settings from the authors as a starting point, a sweep was performed over a wide parameter range. Approximately 500 parameter settings were tried for *MZmine*, and about 50 for *matchedFilter *and *centWave*.

For the parameter optimisation we used the mixture of 14 compounds (MM14). Due to the electrospray ionisation, each compound gives rise to a number of features. A data set of known features was created using the separately measured substances. We annotated features that can be explained as adducts and fragments of the compound as well as their isotopic peaks. For all 14 compounds this results in a set of 296 features, about 21 features per compound. We observed up to eight in-source fragments per compound and also various cluster ions like [2M+H]^+ ^oder [3M+Na]^+^. The annotations are available as Additional file 2. Manual verification shows, that 122 of the 296 known features are clearly visible in the MM14 mixture, while the other 174 features are hard to detect by the human eye. The 122 verified features are considered as required features, which should be detected by the algorithms.

All other features (beyond the 296) which were reported by the programs, but cannot be explained as features originating from the marker mixture, are considered as "false" features, e.g. (usually small) signals from solvents or chemical impurities, background noise etc.

As one result from the optimisation, we found that all algorithms are able to detect more than 100 from the 122 selected real features, but only if approximately 450 "false" features are tolerated. The total number of 122 real features are detected only with settings that give more false positives (see section 3.4). Therefore, as a trade-off between real and "false" features, we chose those parameter settings which detect a maximal number of real features, but return less than 450 "false" features.

Since the algorithms detect around 200–300 features in the separately measured blank solvent, these 450 "false" features can be explained as a "background", consisting of features originating from the solvents, tubes, vials, or impurities of the used marker compounds.

The result of the optimisation process can be seen in Table 2. These parameter settings were used for the following experiments.

**Table 2:** Parameter optimisation using the MM14 marker mixture

Algorithm	Number of detected MM14 features	Number of other reported features	Parameters
centWave	115	443	peakwidth = (5,10), ppm = 30, snthresh = 5, prefilter = (2,400)
matchedFilter	114	425	fwhm = 4, snthresh = 12, step = 0.02, mzdiff = 0, max = 50
MZmine	107	442	bin size = 0.05, chromatographic threshold level = 0.8, intensity tolerance = 0.7, minimum peak duration = 3, minimum peak height = 500, m/z tolerance = 0.03, noise level = 20

Number of features detected in the MM14 marker mixture and the parameter values that were chosen after the parameter optimisation step.

### 2.4 Evaluation

Since feature detection can be seen as an information retrieval task, the performance can be assessed using the *precision *and *recall *values. The recall value (also referred to as *sensitivity*) measures the fraction of relevant items that are found by a query, while the precision value quantifies the relation of relevant items to the false positives. Denoting the total number of features that were detected by an algorithm by *N*, the number of real features that were found by *TP*, and the total number of real features by *NP*, we can measure Recall = $\frac{TP}{NP}$ and Precision = $\frac{TP}{N}$ of a feature detection algorithm. A perfect feature detection algorithm will have both measures equal to 100%. False positives features lower the precision; false negatives (undetected real features) lower the recall.

For a compact representation of the results we used the *F-score *as a combined measure of precision and recall, which is defined as F-score = $\frac{2\cdot R\cdot P}{R+P}$[20]. A perfect feature detection will achieve a F-score of 100%, and both false positives and false negatives features lower its value. The F-score can be interpreted as a measure of the overall performance of a feature detection algorithm.

## Results and discussion

We performed two experiments to assess the performance of the three algorithms. The experiments were designed to evaluate the sensitivity of the algorithms using complex biological samples at different concentrations.

First, the feature set representing the ground truth had to be created. For this purpose we used ten technical replicates of undiluted *Arabidopsis thaliana *seed and leaf extracts from Sample set 2 (solvent/seed/leaf): (0/100/0) and (0/0/100).

Since a manual annotation of the features was out of scope, we applied the following procedure to create a list of reliably detected features:

1. Feature detection on the 2 × 10 samples was performed using the three algorithms

2. We investigated the number of features which are found reproducibly in repeated measurements. The features detected in the ten technical replicates of undiluted seed and leaf extracts were separately aligned using XCMS *group *function (*mzwid *= 0.05, *bw *= 2). After the alignment only those features which were detected in at least seven out of the ten samples were retained. The resulting numbers of features are shown in Table 3.

**Table 3:** Aligned features

	Number of aligned features	
Algorithm	Seed	Leaf
centWave	2634	2423
matchedFilter	1568	1919
MZmine	2529	2699

Number of features that have been reliably detected in at least seven out of ten technical replicates from LC/MS analyses of seed and leaf extracts (Experiment 1).

3. We matched the aligned feature lists of all three algorithms (using 0.015 *m/z *and 5 s tolerance) and removed those features which had been found by only a single algorithm.

**Figure 8:**
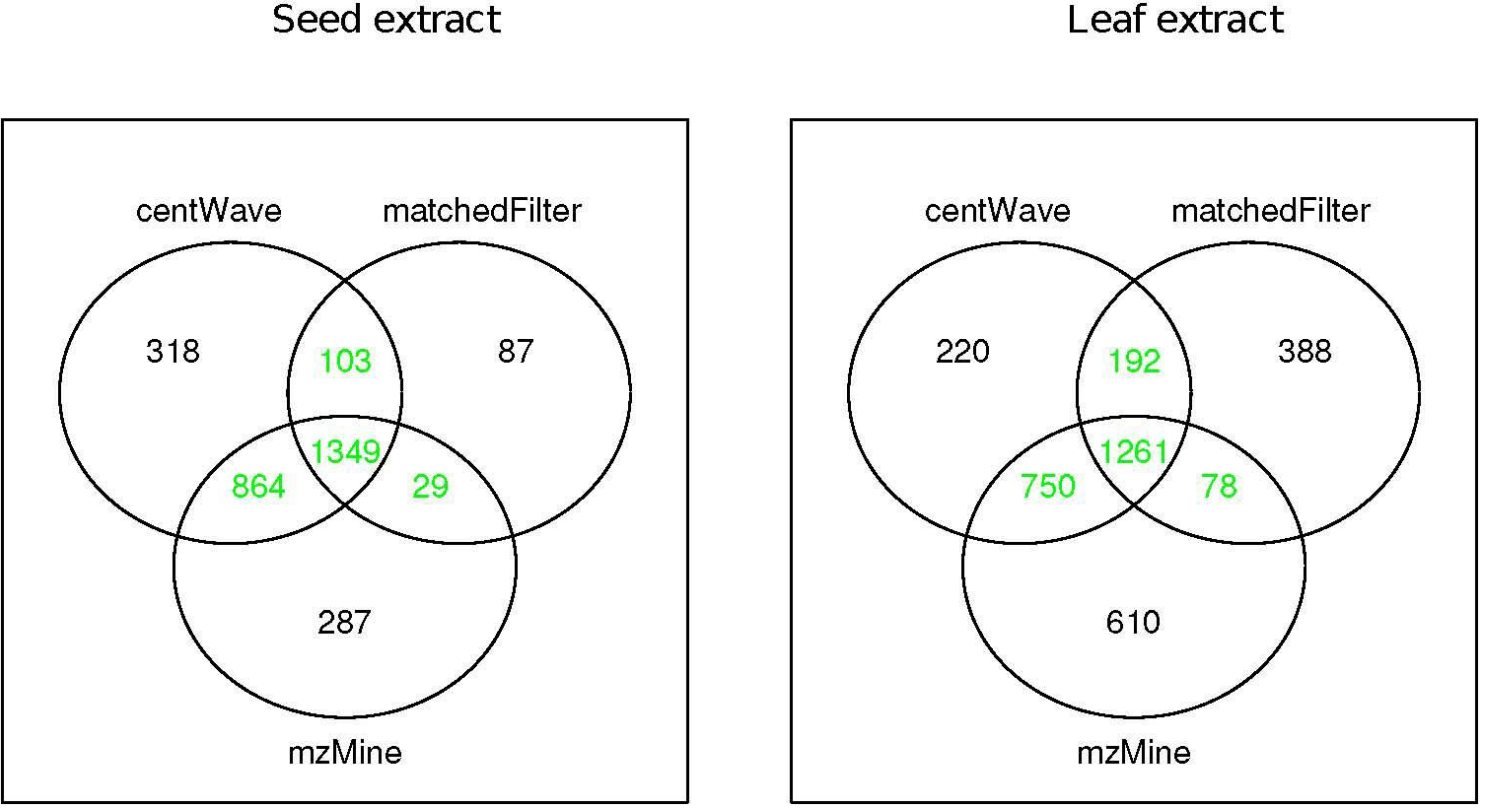
**Venn Diagrams of Detected Features**. Venn Diagrams showing the number of features in seed and leaf extracts that were found by the three different algorithms. Only the overlapping (green coloured) subsets were used as ground truth.

The resulting feature list contains 2281 features for the leaf- and 2345 features for the seed extract. 4076 features are unique, 550 features appear in both extracts. The filtering (step 2. & 3.) retained only the reliable features both across the replicates and detected by the majority of feature detection algorithms, see Figure 8. This data set was considered as ground truth feature data and used for the further evaluation.

### 3.1 Experiment 1

**Figure 9:**
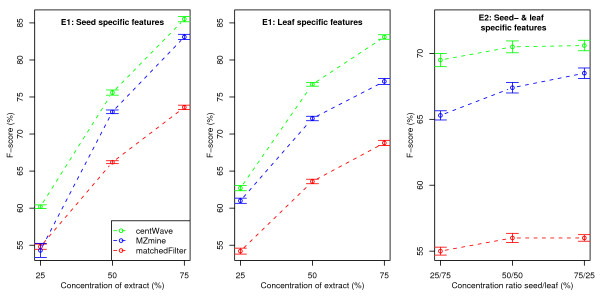
**F-score values for Experiment 1 & 2**. F-score (combined measure of recall and precision, calculated from the ground truth features) for dilution series of the seed and leaf extract (left-most and middle part) and for mixtures of the seed and leaf extract (right-most part of the figure). Detected features that match the respective ground truth features were counted als true positives, while all other features returned were considered as false positives. Higher F-score values represent better feature detection performance.

We evaluated the F-score (calculated from recall and precision values) for dilution series of the seed extract (Sample set 2 (solvent/seed/leaf): (25/75/0), (50/50/0), (75/25/0)). Feature detection was performed on the 3 × 10 samples with the three algorithms using the optimised parameters. Detected features that match the seed specific ground truth features were marked als true positives, while all other returned features were considered as false positives. The results are shown in the the left-most part of Figure 9. The same was done for the leaf specific features and different concentrations of the leaf extract (Sample set 2 (solvent/seed/leaf): (25/0/75), (50/0/50), (75/0/25)). The middle part of Figure 9 depicts the results. The *centWave *algorithm achieved up to 6% higher F-score values than *MZmine *and up to 14% more than *matchedFilter *in this experiment.

### 3.2 Experiment 2

For the second experiment we created mixtures of the seed and leaf extract at different concentrations (Sample set 3) and evaluated the F-score of the ground truth features. Again, feature detection was performed with the three algorithms. The ground truth seed and leaf specific features were considered together as true positives for this measurement. Thereby, the features which appear in both, seed and leaf extracts, were considered only once. All other features that were returned by the algorithms were considered as false positives. The right-most part of Figure 9 shows the result.

The detailed F-score, recall, and precision values of both experiments are available as Additional file 3. By manual inspection of the "true" features that were detected by *centWave*, but not by *MZmine *or *matchedFilter*, we found that these features were often close to other – in many cases larger – chromatographic peaks. This can be interpreted as a masking effect caused by noise level computation on the full chromatogram. The *centWave *algorithm uses local baseline and noise estimation to circumvent this problem.

Looking at the false positive features, we observed that *matchedFilter *frequently reports spikes (very narrow chromatographic peaks, consisting of 1–3 points) while *MZmine *tends to detect features in regions where only a high level noise can be seen.

### 3.3 Runtime

All three algorithms perform the feature detection for one sample in less than two minutes. *centWave *was the fastest algorithm in the test, with on average only one minute runtime per sample. The runtimes shown in Table 4 were measured as wall-clock time including all file input without other programs running. All measurements were done on an AMD Athlon 64 X2 Dual Core Processor 4200+ with 4GB RAM, running Linux (Ubuntu 6.06). Both frameworks can distribute the processing tasks, *MZmine *using Java RMI and XCMS using the Message Passing Interface (MPI) via Rmpi [21] on multicore architectures (and even cluster setups) to speed up the processing of many samples. This option was not used for the runtime measurements.

**Table 4:** Runtimes

	centWave	matchedFilter	MZmine
Runtime in minutes	1.02	1.85	1.54

Average wall-clock runtime in minutes for feature detection in one sample averaged across ten samples containing a 50/50 leaf/seed extract mixture.

### 3.4 Alternative parameter settings

The optimisation strategy outlined in section 2.3 tried to balance the recall and precision, using a rough estimate of the potential chemical background signals. The settings in typical metabolomics profiling experiments of e.g. plant extracts usually will be tuned more aggressively towards higher sensitivity. The resulting false positive features are often filtered by the downstream analysis, such as the alignment of replicate measurements and statistical tests for differential features.

**Figure 10:**
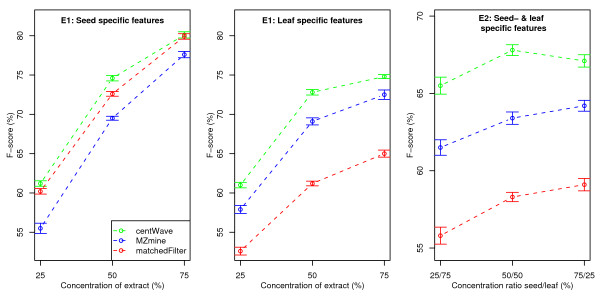
**F-score values for Experiment 1 & 2 (alternative parameter settings)**. F-score (combined measure of recall and precision, calculated from the ground truth features) for dilution series of the seed and leaf extract (left-most and middle part) and for mixtures of the seed and leaf extract (right-most part of the figure). Detected features that match the respective ground truth features were counted als true positives, while all other features returned were considered as false positives. Higher F-score values represent better feature detection performance. Alternative parameter settings were used (see Additional file 4).

We repeated the parameter optimisation, this time allowing up to 1000 background features. Essentially, the respective chromatographic threshold parameters were lowered, to achieve higher sensitivity. With these parameters we recreated the ground truth, and repeated both experiments. The results are depicted in Figure 10. The parameter settings and the number of features in the ground truth, as well as the detailed F-score, recall, and precision values are available as Additional file 4.

With the second parameter set, we observed higher sensitivity for all algorithms. The number of aligned features almost doubled, the resulting ground truth contains 6649 unique features. The recall values of *matchedFilter *improved notably with the alternative parameter settings.

The results based on the second parameter set confirm the general trend shown above. The *centWave *algorithm achieved up to 6% and 15% higher F-score values than *MZmine *and *matchedFilter*, respectively.

## Conclusion

We presented a new feature detection algorithm for high resolution LC/MS data called *centWave*. With the increasing deployment of high-resolution mass spectrometers such as QTOF or Orbitrap instruments, and high-throughput applications such as metabolomics experiments of highly complex samples, a reliable and sensitive feature detection is essential. *centWave *shows a high sensitivity, while trying to keep the false positive features low.

In the past, the Bioconductor project has attracted more and more development related to mass spectrometry and metabolite pathways. The implementation of *centWave *is available in the R-package XCMS and can be obtained from . Integration with Bioconductor provides good support for the common file formats (netCDF, mzData and mzXML, with mzML currently under development) and allows for powerful downstream statistical analysis. The user feedback on the XCMS mailing list showed, that the *centWave *algorithm (introduced in 2007) is successfully used for LC-QTOF, LC-Orbitrap and even CE-MS or GC-MS data. For a more objective comparison we have evaluated *centWave *against two other open source algorithms. We performed two experiments to assess the performance of the algorithms, using complex chemical mixtures at different concentrations. The F-score, as a combined measure of recall and precision, was calculated using the ground truth data. The result was for *centWave *always higher than for *matchedFilter *and *MZmine*. The *centWave *algorithm is based on a sensitive detection of potentially interesting mass traces (ROIs), followed by an extensive chromatographic analysis, that reliably detects chromatographic peaks with different width via CWT. To allow for high sensitivity, baseline and noise are estimated locally. Some efforts are made to locate the exact chromatographic peak boundaries to provide accurate peak intensities. Feature quality can be assessed using numerous metrics, including signal to noise ratio, m/z fluctuation, and the residual of the Gaussian fit. Further development of the *centWave *algorithm will include an automatic estimation of the processing parameters.

In addition to *centWave *and the LC/MS data sets we have released the manual annotation of an LC/MS measurement of several pure compounds as a benchmark data set for both machine and software comparisons. The data sets are available at .

## Authors contributions

CB performed the LC/MS measurements and was involved in the development of the *centWave*. RT designed and implemented the *centWave *algorithm. RT and SN performed the evaluation of the algorithms. All authors contributed to, read and approved the fnal manuscript.

## Supplementary Material

Additional file 1 **Experimental setup**. Detailed description of materials, chemicals, and protocols. Click here for file

Additional file 2 **MM14 annotations**. Feature annotations for the mixture of 14 compounds (MM14). Click here for file

Additional file 3 **Detailed recall, precision, and F-score values**. Tables containing the detailed F-score, recall, and precision values of both experiments. Click here for file

Additional file 4 **Results for the alternative parameter settings**. Venn diagrams of the ground truth data as well as the detailed F-score, recall, and precision values of both experiments using alternative parameter settings. Click here for file
