# Supplementary material for: Highly sensitive feature detection for high resolution LC/MS
Source: BMC Bioinformatics. 2008 Nov 28;9:504. doi: 10.1186/1471-2105-9-504 (PMC2639432; doi:10.1186/1471-2105-9-504)
Supplement: Additional file 1 — Experimental setup. Detailed description of materials, chemicals, and protocols. [file 1471-2105-9-504-S1.pdf]

Additional File 1
for
Highly sensitive feature detection for high resolution LC/MS
by

Ralf Tautenhahn, Christoph Böttcher, and Steffen Neumann

Experimental setup

Chemicals and Materials

All solvents used were of LC/MS grade quality. o-Anisic acid, biochanin A, p-coumaric acid, ferulic acid, N-(3-indolylacetyl)-L-valine and kinetin were obtained from Sigma-Aldrich. Indole-3-acetonitrile, indole-3-carbaldehyde, kaempferol, phloretin, phlorizin dihydrate and phenylglycine were purchased from Fluka, rutin trihydrate from Acros and phenylalanine-d5 from Cambridge Isotope Laboratories. Methanolic stock solutions (5 mM) of each compound were prepared, except phenylglycine and phenylalanine-d5, which were dissolved in water/methanol/formic acid, 69.75/29.75/0.5 (v/v/v). *Arabidopsis thaliana* (ecotype Col-0) was grown in a phyto-chamber on a soil/vermiculite mixture (3/2) at 22C, 70% relative humidity and an photoperiod of 8 h per day at 100 $\mu Em^{-2}s^{-1}$. Rosette leaves of twenty plants were partially harvested after 6 weeks (growth stage 3.5-3.7) and pooled. Afterwards, plants were transferred to a fully climatized green house and grown at 22C and 60% relative humidity under a photoperiod of 16 h per day until final seed set (growth stage 9.7). Seeds of twenty plants were harvested and pooled.
Preparation of leaf extracts
Freshly ground *Arabidopsis thaliana* leaf tissue (100 ± 5 mg) was subjected twice to the following extraction procedure: mixing with 200 μL of methanol/water, 4/1 (v/v), sonication at 22C for 15 min and centrifugation for 10 min. Both extracts were combined and evaporated to dryness in a vacuum centrifuge at ambient temperature. The remaining residue was redissolved in 400 μL methanol/water, 3/7 (v/v), and filtered through a 0.2 μm PTFE syringe filter. Ten extracts were prepared from the leaf pool and combined.

Preparation of seed extracts

Arabidopsis thaliana seeds (10 ± 0.5 mg) were homogenized in 1000 μL methanol/water, 80/20 (v/v) using 0.3 g zirconia beeds (BioSpec Products) in a MiniBeadBeater (BioSpec Products). After centrifugation 750 μL of the supernatant were evaporated to dryness in a vacuum centrifuge at ambient temperature. The remaining residue was redissolved in 750 μL methanol/water, 30/70 (v/v) and filtered through a 0.2 μm PTFE syringe filter. Ten extracts were prepared from the seed pool and combined.

UPLC/ESI-QTOF-MS

Chromatographic separations were performed on an Acquity UPLC system (Waters) equipped with a modified C_{18} column (HSS T3, 1.0 x 100 mm, particle size 1.8 μm, Waters) applying the following binary gradient at a flow rate of 150 μL min^{-1}: 0-1 min, isocratic 95% A (water, 0.1% formic acid), 5% B (acetonitrile, 0.1% formic acid); 1-16 min, linear from 5 to 95% B; 16-18 min, isocratic 95% B; 18-20 min, isocratic 5% B. The injection volume was 2 μL. Eluted compounds were detected from m/z 100-1000 by a MicrOTOF-Q hybrid quadropole time-of-flight mass spectrometer (Bruker Daltonics) equipped with an Apollo II electrospray ion source in positive ion mode using following instrument settings: nebulizer gas, nitrogen, 1.6 bar; dry gas, nitrogen, 6 L/min, 190C; collision gas, argon; capillary, -6000 V; end plate offset, -500 V; collision RF 200 Vpp (50%)/400 Vpp (50%), transfer time, 70 μs; pre pulse storage, 5 μs; pulser frequency, 10 kHz; spectra rate, 3 Hz. Mass calibration was achieved by injection of 10 mM lithium formiate in isopropanol/water, 1/1 (v/v) at the end of the chromatographic gradient using a diverter valve and a separate pump.
