# Supplementary material for: Highly sensitive feature detection for high resolution LC/MS
Source: BMC Bioinformatics. 2008 Nov 28;9:504. doi: 10.1186/1471-2105-9-504 (PMC2639432; doi:10.1186/1471-2105-9-504)
Supplement: Additional file 2 — MM14 annotations. Feature annotations for the mixture of 14 compounds (MM14). [file 1471-2105-9-504-S2.pdf]

Additional File 2
for
Highly sensitive feature detection for high resolution LC/MS
by
Ralf Tautenhahn, Christoph Böttcher, Steffen Neumann

1 MM14 annotations

```
#
# MM14 Spectra, Bruker micrOTOF-Q
#

# Format:
# Compound, retention time (in seconds)
# Peak Annotations
# Calculated m/z values
# Observed relative intensities


' o-Anisic acid', 280
[C6H5]+, [C6H5+1]+,  [C6H7]+, [C6H7+1]+,  [C6H4O]+, [C6H4O+1]+,  [C7H5O]+, [C7H5O+1]+,
[C7H7O]+, [C7H7O+1]+,  [C7H4O2]+, [C7H4O2+1]+,  [C8H7O2]+, [C8H7O2+1]+, [C8H7O2+2]+,
[M+H]+, [M+H+1]+,  [M+Na]+, [M+Na+1]+,  [M+K]+, [M+K+1]+, [M+2Na-H]+, [M+2Na-H+1]+,
[3M+K+H]2+, [3M+K+H+1]2+,  [2M+Na]+, [2M+Na+1]+,  [2M+K]+, [2M+K+1]+, [2M+3Na-2H]+,
[2M+3Na-2H+1]+,  [3M+K]+, [3M+K+1]+,  [3M+4Na-3H]+, [3M+4Na-3H+1]+,  [3M+Na+K-2H]+,
[3M+Na+K-2H+1]+
77.03858, 78.04725,  79.05423, 80.06290,  92.02567, 93.03434,  105.0334, 106.042155,
 107.0491, 108.05781,  120.02058, 121.02925,  135.04406, 136.05273, 137.06139,
153.05462, 154.06329,  175.03657, 176.04524,  191.0105, 192.01917,  197.01851,
198.02718,   248.0562, 248.56057,  327.08391, 328.09258,  343.05785, 344.06652,
371.0478, 372.05647,  495.1052, 496.11387,  545.0771, 546.08577,  555.0430,
556.05167
20, 1,  2, 0,   5, 0,   1, 0,  1, 0,  1, 0,  100, 9, 1,  9, 1,  2,
 0,  24, 3,  6, 1,  5, 2,  1, 0,  3, 1,  2, 1,  2, 1,  1, 1,  1, 0


'biochanin A', 540
[C14H13O3]+, [C14H13O3+1]+,  [C15H9O4]+, [C15H9O4+1]+,  [C15H10O5]+, [C15H10O5+1]+,
[M+H]+, [M+H+1]+, [M+H+2]+, [M+Na]+, [M+Na+1]+, [M+Na+2]+,  [3M+K+H]2+, [3M+K+H+1]2+,
[2M+H]+, [2M+H+1]+,  [2M+Na]+, [2M+Na+1]+,  [2M+K]+, [2M+K+1]+,  [3M+Na]+, [3M+Na+1]+,
[3M+K]+, [3M+K+1]+
229.08592, 230.094585,  253.04954, 254.05821,  270.05227, 271.06094,  285.07575,
286.08442, 287.09308,  307.05769, 308.06636, 309.07502,  446.0880, 447.09667,
569.14422, 570.15289,  591.12617, 592.13484,   607.10011, 608.10878,  875.1947,
876.20337,  891.1687, 892.17737
0, 0,  0, 0,  0, 0,  100, 20, 3,  16, 3, 1,  0, 0,  0, 0,  1, 0,  2, 1,  0, 0,  1, 0


'ferulic acid', 217
[C7H5]+, [C7H5+1]+,  [C8H5O]+, [C8H5O+1]+,  [C9H5O2]+, [C9H5O2+1]+,  [C9H9O2]+,
[C9H9O2+1]+,  [C10H9O3]+, [C10H9O3+1]+, [C10H9O3+2]+,  [M+H]+, [M+H+1]+, [2M+K+H]2+,
[2M+K+H+1]2+, [M+Na]+, [M+Na+1]+,  [M+K]+, [M+K+1]+,  [3M+K+H]2+, [3M+K+H+1]2+,
[2M+K]+, [2M+K+1]+, [3M+Na]+, [3M+Na+1]+,  [3M+K]+, [3M+K+1]+
89.03858, 90.04725,  117.03349, 118.042155, 145.02841, 146.037075,  149.05971,
150.06838,  177.05462, 178.06329, 179.07195, 195.06519, 196.07386,  214.0431,
```

215.05177, 217.04713, 218.0558, 233.02107, 234.02974, 311.0672, 312.07587,
427.0790, 428.08767, 605.1629, 606.17157, 621.1369, 622.14557
2, 0, 8, 1, 34, 3, 5, 1, 100, 10, 1, 16, 2, 4, 1, 16, 2, 3, 1, 5, 1,
10, 3, 4, 2, 8, 2

'N-(3-indolylacetyl)-L-valine', 382
[C4H10N]+, [C4H10N+1]+, [C5H12NO2]+, [C5H12NO2+1]+, [C9H8N]+, [C9H8N+1]+,
[C10H8NO]+, [C10H8NO+1]+, [M+H-H2O]+, [M+H-H2O+1]+, [M+H]+, [M+H+1]+, [M+H+2]+,
[M+Na]+, [M+Na+1]+, [M+H+CH3CN]+, [M+H+CH3CN+1]+, [2M+Na]+, [2M+Na+1]+, [2M+K]+,
[2M+K+1]+, [2M+2Na-H]+, [2M+2Na-H+1]+, [3M+Na]+, [3M+Na+1]+, [3M+K]+, [3M+K+1]+
72.08078, 73.08945, 118.08626, 119.09493, 130.06513, 131.0738, 158.06004,
159.06871, 258.13573, 259.144395, 275.13902, 276.14769, 277.15635, 297.1210,
298.12967, 316.1656, 317.17427, 571.2527, 572.26137, 587.2266, 588.23527,
593.2346, 594.24327, 845.3844, 846.39307, 861.3584, 862.36707
1, 0, 4, 0, 12, 1, 0, 0, 1, 0, 100, 18, 3, 1, 0, 1, 0, 3, 1, 5, 2,
1, 0, 1, 1, 3, 1

'indole-3-acetonitrile', 400
[C8H7N]+, [C8H8N]+, [C9H8N]+, [C9H8N+1]+, [C9H8N+2]+, [C10H7N2]+, [C10H7N2+1]+,
[M+H]+, [M+H+1]+, [M+Na]+, [M+Na+1]+, [3M+H+K]2+, [3M+H+K+1]2+, [4M+H+K]2+,
[4M+H+K+1]2+, [2M+Na]+, [2M+Na+1]+
117.0573, 118.06513, 130.06513, 131.0738, 132.08246, 155.06037, 156.06904,
157.07602, 158.08469, 179.05797, 180.06664, 254.0883, 255.09697, 332.1226,
333.13127, 335.1266, 336.13527
31, 4, 100, 11, 1, 22, 4, 11, 2, 7, 1, 6, 0, 3, 1, 2, 1

'indole-3-carbaldehyde', 300
[C7H7]+, [C7H7+1]+, [C8H7N]+, [C8H8N]+, [C8H8N+1]+, [C8H8N+2]+, [C9H6NO+],
[C9H7NO]+, [C9H8NO]+, [C9H8NO+1]+, [C9H7NNaO]+, [C9H7NNaO+1]+
91.05423, 92.062895, 117.0573, 118.06513, 119.073795, 120.08246, 144.04439,
145.05222, 146.06004, 147.06871, 168.04198, 169.05065
23, 2, 8, 100, 17, 1, 2, 2, 35, 3, 7, 1

'kaempferol', 424
[M+H]+, [M+H+1]+, [M+H+2]+, [M+Na]+, [M+Na+1]+, [2M+K]+, [2M+K+1]+,
[3M+Na]+, [3M+Na+1]+
287.05501, 288.06368, 289.07234, 309.0369, 310.04557, 611.0586, 612.06727,
881.1323, 882.14097
100, 20, 3, 4, 1, 1, 0, 0, 0

'kinetin', 193
[C5H5O]+, [C5H5O+1]+, [C5H3N4]+, [C5H3N4+1]+, [C5H6N5]+, [C5H6N5+1]+,
[C6H6N5]+, [C6H6N5+1]+, [C9H7N4]+, [C9H7N4+1]+, [C8H7N5]+, [C8H7N5+1]+,
[C9H10N5]+, [C9H10N5+1]+, [C10H8N5]+, [C10H8N5+1]+, [M+H]+, [M+H+1]+, [M+H+2]+,
[M+Na]+, [M+Na+1]+, [2M+Na]+, [2M+Na+1]+
81.03349, 82.042155, 119.03522, 120.04389, 136.06177, 137.07044, 148.06177,
149.07044, 171.06652, 172.07519, 173.0696, 174.07827, 188.09307, 189.10174,
198.07742, 199.08609, 216.08799, 217.09666, 218.10532, 238.0699, 239.07857,
453.1506, 454.15927
58, 4, 1, 0, 5, 1, 30, 2, 1, 0, 7, 2, 23, 2, 2, 0, 100, 24, 1, 5, 0, 1, 0

'p-coumaric acid', 257

[C7H7]+, [C7H7+1]+, [C8H7O]+, [C8H7O+1]+, [C9H7O2]+, [C9H7O2+1]+, [C9H7O2+2]+, [M+H]+, [M+H+1]+, [M+Na]+, [M+Na+1]+, [M+K]+, [M+K+1]+, [2M+K]+, [2M+K+1]+, [3M+K]+, [3M+K+1]+
91.05423, 92.062895, 119.04914, 120.05781, 147.04406, 148.05273, 149.06139, 165.05462, 166.06329, 187.03657, 188.04524, 203.0105, 204.01917, 367.0578, 368.06647, 531.1051, 532.11377
21, 1, 55, 5, 100, 10, 1, 20, 1, 10, 3, 11, 1, 29, 5, 6, 3

'phenylalanine - d5', 82
[C8H6D4N]+, [C8H5D5N]+, [C8H5D5N+1]+, [C9H7D5NO2]+, [C9H7D5NO2+1]+
124.10588, 125.11455, 126.12321, 171.11764, 172.12631
21, 100, 11, 8, 1

'phenylglycine', 43
[C6H5]+, [C6H5+1]+, [C6H7]+, [C6H7+1]+, [C7H8N]+, [C7H7O]+, [C7H7O+1]+, [C8H7O2]+, [C8H7O2+1]+, [M+H]+, [M+H+1]+, [M+Na]+, [M+Na+1]+, [2M+H]+, [2M+H+1]+, [2M+Na]+, [2M+Na+1]+
77.03858, 78.04725, 79.05423, 80.062895, 106.06513, 107.04914, 108.05781, 135.04406, 136.05273, 152.0706, 153.07927, 174.05255, 175.06122, 303.13393, 304.1426, 325.1158, 326.1245
34, 2, 82, 9, 7, 74, 9, 100, 9, 13, 1, 7, 1, 2, 0, 6, 1

'phloretin', 415
[C7H7O]+, [C7H7O+1]+, [C6H7O3]+, [C6H7O3+1]+, [C9H9O2]+, [C9H9O2+1]+, [C8H7O3]+, [C8H7O3+1]+, [C7H5O4]+, [C7H5O4+1]+, [C8H9O4]+, [C8H9O4+1]+, [C9H9O4]+, [C9H9O4+1]+, [C15H14O4], [C15H14O4+1]+, [M+H]+, [M+H+1]+, [M+H+2]+, [M+Na]+, [M+Na+1]+, [M+K]+, [M+K+1]+, [3M+H+K]2+, [3M+H+K+1]2+, [2M+Na]+, [2M+Na+1]+, [2M+K]+, [2M+K+1]+
107.04914, 108.05781, 127.03897, 128.04764, 149.05971, 150.06838, 151.03897, 152.04764, 153.01824, 154.02691, 169.04954, 170.05821, 181.04954, 182.05821, 258.08866, 259.09733, 275.0914, 276.10007, 277.10873, 297.0733, 298.08197, 313.0473, 314.05597, 431.10822, 431.61255, 571.1574, 572.16607, 587.1314, 588.14007
86, 8, 2, 0, 3, 0, 1, 0, 1, 0, 62, 5, 2, 0, 1, 0, 100, 18, 2, 41, 6, 2, 0, 8, 4, 7, 3, 31, 12

'phlorizin', 327
[C7H7O]+, [C7H7O+1]+, [C6H7O3]+, [C6H7O3+1]+, [C9H9O2]+, [C9H9O2+1]+, [C8H9O4]+, [C8H9O4+1]+, [C15H15O5]+, [C15H15O5+1]+, [C15H15O5+2]+, [M+H]+, [M+H+1]+, [2M+H+Na]2+, [2M+H+Na+1]2+, [2M+H+K]2+, [2M+H+K+1]2+, [M+Na]+, [M+Na+1]+, [M+K]+, [M+K+1]+, [3M+H+Na]2+, [3M+H+Na+1]2+, [3M+H+K]2+, [3M+H+K+1]2+, [4M+H+K]2+, [4M+H+K+1]2+, [2M+Na]+, [2M+Na+1]+, [2M+K]+, [2M+K+1]+
107.04914, 108.05781, 127.03897, 128.04764, 149.05971, 150.06838, 169.04954, 170.05821, 275.0914, 276.10007, 277.10873, 437.14422, 438.15289, 448.1352, 448.63953, 456.1222, 456.62653, 459.1270, 460.1357, 475.1002, 476.10887, 666.2037, 666.70803, 674.1907, 674.69503, 892.2592, 892.76353, 895.2632, 895.76753, 911.2372, 911.74153
42, 5, 1, 0, 1, 0, 33, 3, 100, 18, 2, 18, 3, 1, 0, 30, 18, 78, 18, 3, 1, 1, 1, 25, 18, 9, 9, 4, 0, 1, 0

'rutin', 273
[C4H5O2]+, [C4H5O2+1]+, [C6H9O3]+, [C6H9O3+1]+, [C6H11O4]+, [C6H11O4+1]+, [C15H11O7]+, [C15H11O7+1]+, [C15H11O7+2]+, [C21H21O12]+, [C21H21O12+1]+, [M+H]+, [M+H+1]+, [2M+H+K]2+, [2M+H+K+1]2+, [M+Na]+, [M+Na+1]+, [2M+2K]2+, [2M+2K+1]2+, [3M+H+K]2+, [3M+H+K+1]2+
85.0284, 86.03707, 129.05462, 130.06329, 147.06519, 148.07386, 303.04993, 304.0586, 305.06726, 465.10275, 466.11142, 611.16066, 612.16933, 630.1386, 630.64293, 633.14261,

```
 634.15128, 649.1166, 649.62093,  935.2153, 935.71963
1, 0,  3, 0,  1, 0,  100, 27, 5,  85, 27,  73, 29,  27, 15,  32, 12,  1, 0,  4, 3
```
