# Supplementary material for: Highly sensitive feature detection for high resolution LC/MS
Source: BMC Bioinformatics. 2008 Nov 28;9:504. doi: 10.1186/1471-2105-9-504 (PMC2639432; doi:10.1186/1471-2105-9-504)
Supplement: Additional file 3 — Detailed recall, precision, and F-score values. Tables containing the detailed F-score, recall, and precision values of both experiments. [file 1471-2105-9-504-S3.pdf]

Additional File 3
for
Highly sensitive feature detection for high resolution LC/MS
by

Ralf Tautenhahn, Christoph Böttcher, Steffen Neumann

1 Results using optimised parameter settings

1.1 Dilution series of seed and leaf extracts

Ratio of concentrations (%)			F-score values for the ground truth seed and leaf features (%)		
Solvent	Seed	Leaf	centWave	matchedFilter	MZmine
75	25	0	60.2 ± 0.5	54.8 ± 0.7	54.3 ± 1.9
50	50	0	75.6 ± 0.7	66.2 ± 0.4	73 ± 0.5
25	75	0	85.5 ± 0.7	73.6 ± 0.6	83.1 ± 0.7
75	0	25	62.7 ± 0.7	54.2 ± 0.8	61 ± 0.7
50	0	50	76.7 ± 0.5	63.6 ± 0.6	72.1 ± 0.6
25	0	75	83.1 ± 0.6	68.8 ± 0.7	77.1 ± 0.8

Table 1: F-score values for seed (upper part) and leaf (lower part) ground truth features measured for leaf and seed extracts in solvent. The numbers represent the average F-score value across ten technical replicates in percent and their standard deviation. The F-score was calculated using the recall values in Table 2 and the precision values in Table 3.

Ratio of concentrations (%)			Recall values for the ground truth seed and leaf features (%)		
Solvent	Seed	Leaf	centWave	matchedFilter	MZmine
75	25	0	49.9 ± 0.4	40.6 ± 0.6	56.3 ± 1.1
50	50	0	71.8 ± 0.6	52.8 ± 0.4	67.2 ± 0.6
25	75	0	90 ± 1.2	62 ± 0.6	84.8 ± 1.4
75	0	25	52 ± 0.7	43.9 ± 0.7	50.1 ± 0.7
50	0	50	73.2 ± 0.9	56.3 ± 0.6	69.8 ± 0.5
25	0	75	88.2 ± 0.9	65.1 ± 0.8	84.7 ± 0.8

Table 2: Recall values for seed (upper part) and leaf (lower part) ground truth features measured for leaf and seed extracts in solvent. The numbers represent the average recall value across ten technical replicates in percent and their standard deviation.

Ratio of concentrations (%)			Precision values for the ground truth seed and leaf features (%)		
Solvent	Seed	Leaf	centWave	matchedFilter	MZmine
75	25	0	75.9 ± 1.4	84.1 ± 1	52.4 ± 2.6
50	50	0	79.8 ± 1	88.5 ± 0.6	79.9 ± 0.7
25	75	0	81.5 ± 0.8	90.4 ± 0.7	81.5 ± 0.6
75	0	25	79 ± 1.5	70.7 ± 1	77.9 ± 1.5
50	0	50	80.6 ± 1.1	73.2 ± 0.7	74.5 ± 1.1
25	0	75	78.7 ± 1	73 ± 0.8	70.6 ± 1

Table 3: Precision values for seed (upper part) and leaf (lower part) ground truth features measured for leaf and seed extracts in solvent. The numbers represent the average precision value across ten technical replicates in percent and their standard deviation.

1.2 Mixtures of seed and leaf extracts

Ratio of concentrations (%)			F-score values for the ground truth seed and leaf features (%)		
Solvent	Seed	Leaf	centWave	matchedFilter	MZmine
0	25	75	69.5 ± 1	55 ± 0.6	65.3 ± 0.7
0	50	50	70.5 ± 0.9	56 ± 0.7	67.4 ± 0.8
0	75	25	70.6 ± 0.8	56 ± 0.5	68.5 ± 0.8

Table 4: F-score values for seed and leaf ground truth features (set union) measured for mixtures of leaf and seed extracts. The numbers represent the average F-score value across ten technical replicates in percent and their standard deviation. The F-score was calculated using the recall values in Table 5 and the precision values in Table 6.

Ratio of concentrations (%)			Recall values for the ground truth seed and leaf features (%)		
Solvent	Seed	Leaf	centWave	matchedFilter	MZmine
0	25	75	61.5 ± 1	42.6 ± 0.5	58.7 ± 0.7
0	50	50	62.5 ± 0.7	42.7 ± 0.7	60 ± 0.8
0	75	25	62.1 ± 1.1	41.7 ± 0.7	59.5 ± 1.1

Table 5: Recall values for seed and leaf ground truth features (set union) measured for mixtures of leaf and seed extracts. The numbers represent the average recall value across ten technical replicates in percent and their standard deviation.

Ratio of concentrations (%)			Precision values for the ground truth seed and leaf features (%)		
Solvent	Seed	Leaf	centWave	matchedFilter	MZmine
0	25	75	79.9 ± 0.9	77.5 ± 1.1	73.5 ± 1.1
0	50	50	80.9 ± 1.3	81.3 ± 0.9	76.8 ± 1
0	75	25	81.7 ± 0.8	85.1 ± 1	80.7 ± 0.9

Table 6: Precision values for seed and leaf ground truth features (set union) measured for mixtures of leaf and seed extracts. The numbers represent the average precision value across ten technical replicates in percent and their standard deviation.

Ratio of concentrations (%)			Recall values for the ground truth seed and leaf features (%)		
Solvent	Seed	Leaf	centWave	matchedFilter	MZmine
0	25	75	47.6 ± 1.1	32.8 ± 0.7	45.1 ± 0.9
0	50	50	63.6 ± 1.5	41.9 ± 1.3	60.7 ± 1.4
0	75	25	79 ± 2.4	50.4 ± 1.8	75.7 ± 2.3
0	75	25	53.4 ± 0.9	40.3 ± 1	51.6 ± 0.7
0	50	50	69.9 ± 0.6	51.1 ± 0.8	68 ± 0.8
0	25	75	84.6 ± 1.1	60.7 ± 0.4	81.7 ± 0.5

Table 7: Recall values for seed (upper part) and leaf (lower part) ground truth features measured for mixtures of leaf and seed extracts. The numbers represent the average recall value across ten technical replicates in percent and their standard deviation.
