# Supplementary material for: Highly sensitive feature detection for high resolution LC/MS
Source: BMC Bioinformatics. 2008 Nov 28;9:504. doi: 10.1186/1471-2105-9-504 (PMC2639432; doi:10.1186/1471-2105-9-504)
Supplement: Additional file 4 — Results for the alternative parameter settings. Venn diagrams of the ground truth data as well as the detailed F-score, recall, and precision values of both experiments using alternative parameter settings. [file 1471-2105-9-504-S4.pdf]

Additional File 4
for
Highly sensitive feature detection for high resolution LC/MS
by

Ralf Tautenhahn, Christoph Böttcher, Steffen Neumann

1 Results using alternative parameter settings

1.1 Parameter optimisation using the MM14 marker mixture

Algorithm	Number of detected MM14 features	Number of other reported features	Parameters
centWave	136	898	peakwidth=(5,10), ppm=30, snthresh=4, prefilter=(2,200)
matchedFilter	144	917	fwhm=4, snthresh=7, step=0.025, mzdiff=0, max=50
MZmine	124	907	bin size=0.05, chromatographic threshold level=0.85, intensity tolerance=0.7, minimum peak duration=3, minimum peak height=300, m/z tolerance=0.03, noise level=20

Table 1: Number of features detected in the MM14 marker mixture and the parameter values that were chosen after the parameter optimisation step.

1.2 Creation of ground truth

1.2.1 Aligned features

	Number of aligned features	
Algorithm	Seed	Leaf
centWave	4419	3854
matchedFilter	3286	4250
MZmine	4025	4171

Table 2: Number of features that have been reliably detected in at least seven out of ten technical replicates from LC/MS analyses of seed and leaf extracts (Experiment 1).

1.2.2 Venn diagrams

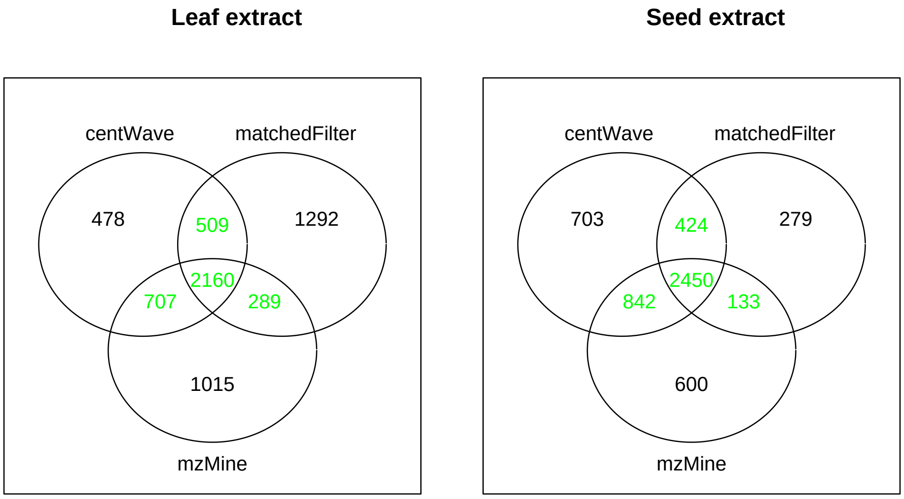

Figure 1: Venn Diagrams showing the number of features in leaf and extracts that were found by the three different algorithms. Only the overlapping (green coloured) subsets were used as ground truth.

1.3 Dilution series of seed and leaf extracts

Ratio of concentrations (%)			F-score values for the ground truth seed and leaf features (%)		
Solvent	Seed	Leaf	centWave	matchedFilter	MZmine
75	25	0	61.2 ± 0.7	60.2 ± 0.7	55.5 ± 1.3
50	50	0	74.6 ± 0.7	72.6 ± 0.6	69.5 ± 0.5
25	75	0	80.1 ± 0.8	79.9 ± 0.7	77.6 ± 0.8
75	0	25	61 ± 0.7	52.6 ± 1	57.9 ± 1
50	0	50	72.8 ± 0.7	61.2 ± 0.6	69.1 ± 0.9
25	0	75	74.8 ± 0.5	65 ± 0.9	72.5 ± 1.2

Table 3: F-score values for seed (upper part) and leaf (lower part) ground truth features measured for leaf and seed extracts in solvent. The numbers represent the average F-score value across ten technical replicates in percent and their standard deviation. The F-score was calculated using the recall values in Table 4 and the precision values in Table 5.

Ratio of concentrations (%)			Recall values for the ground truth seed and leaf features (%)		
Solvent	Seed	Leaf	centWave	matchedFilter	MZmine
75	25	0	52.6 ± 0.6	48.7 ± 0.6	45.5 ± 0.8
50	50	0	73.7 ± 0.7	64.4 ± 0.5	65.1 ± 0.6
25	75	0	87.7 ± 1.1	75.5 ± 0.6	80.2 ± 1.1
75	0	25	53.5 ± 0.8	49.4 ± 0.9	49.7 ± 0.7
50	0	50	73.4 ± 0.9	65.1 ± 0.5	69.1 ± 0.6
25	0	75	84.1 ± 1	75.8 ± 0.9	80.9 ± 0.8

Table 4: Recall values for seed (upper part) and leaf (lower part) ground truth features measured for leaf and seed extracts in solvent. The numbers represent the average recall value across ten technical replicates in percent and their standard deviation.

Ratio of concentrations (%)			Precision values for the ground truth seed and leaf features (%)		
Solvent	Seed	Leaf	centWave	matchedFilter	MZmine
75	25	0	73.2 ± 1.2	78.7 ± 1	71.1 ± 2.8
50	50	0	75.5 ± 0.8	83.1 ± 1	74.6 ± 0.8
25	75	0	73.7 ± 1	84.9 ± 1.1	75.2 ± 0.9
75	0	25	71 ± 0.9	56.3 ± 1	69.4 ± 2.1
50	0	50	72.2 ± 0.8	57.7 ± 0.7	69.2 ± 1.4
25	0	75	67.4 ± 0.7	56.9 ± 0.9	65.7 ± 1.5

Table 5: Precision values for seed (upper part) and leaf (lower part) ground truth features measured for leaf and seed extracts in solvent. The numbers represent the average precision value across ten technical replicates in percent and their standard deviation.

1.4 Mixtures of seed and leaf extracts

Ratio of concentrations (%)			F-score values for the ground truth seed and leaf features (%)		
Solvent	Seed	Leaf	centWave	matchedFilter	MZmine
0	25	75	65.5 ± 1.1	55.8 ± 1.1	61.5 ± 1
0	50	50	67.8 ± 0.7	58.3 ± 0.6	63.4 ± 0.8
0	75	25	67.1 ± 0.8	59.1 ± 0.8	64.2 ± 0.7

Table 6: F-score values for seed and leaf ground truth features (set union) measured for mixtures of leaf and seed extracts. The numbers represent the average F-score value across ten technical replicates in percent and their standard deviation. The F-score was calculated using the recall values in Table 7 and the precision values in Table 8.

Ratio of concentrations (%)			Recall values for the ground truth seed and leaf features (%)		
Solvent	Seed	Leaf	centWave	matchedFilter	MZmine
0	25	75	59.8 ± 0.9	49.7 ± 1	55 ± 0.7
0	50	50	62 ± 0.8	50.3 ± 0.7	56.6 ± 0.7
0	75	25	61 ± 1.1	49.1 ± 1	56.2 ± 0.9

Table 7: Recall values for seed and leaf ground truth features (set union) measured for mixtures of leaf and seed extracts. The numbers represent the average recall value across ten technical replicates in percent and their standard deviation.

Ratio of concentrations (%)			Precision values for the ground truth seed and leaf features (%)		
Solvent	Seed	Leaf	centWave	matchedFilter	MZmine
0	25	75	72.3 ± 1.3	63.6 ± 1.5	69.7 ± 1.7
0	50	50	74.8 ± 0.8	69.3 ± 0.8	72.2 ± 1.3
0	75	25	74.6 ± 0.7	74.2 ± 0.6	75.1 ± 1.1

Table 8: Precision values for seed and leaf ground truth features (set union) measured for mixtures of leaf and seed extracts. The numbers represent the average precision value across ten technical replicates in percent and their standard deviation.

Ratio of concentrations (%)			Recall values for the ground truth seed and leaf features (%)		
Solvent	Seed	Leaf	centWave	matchedFilter	MZmine
0	25	75	47.7 ± 1.2	38.1 ± 1.2	41.9 ± 0.9
0	50	50	63.1 ± 1.7	50.6 ± 1.3	56.5 ± 1
0	75	25	76.4 ± 2.2	62 ± 2.3	70.7 ± 2.1
0	75	25	52.7 ± 1	44 ± 1.1	49.6 ± 0.9
0	50	50	68.7 ± 0.9	58.3 ± 1.2	65.3 ± 1.2
0	25	75	80.9 ± 0.8	70.5 ± 0.9	77.8 ± 0.6

Table 9: Recall values for seed (upper part) and leaf (lower part) ground truth features measured for mixtures of leaf and seed extracts. The numbers represent the average recall value across ten technical replicates in percent and their standard deviation.
